# State Assistance to Voluntary Hospitals. Free Donations from Income- and Super-Taxes

**Published:** 1920-04-17

**Authors:** R. H. Caird

**Affiliations:** Chairman of the Board of Management. London Homœopathic Hospital, W.C. 1


### THE EDITOR'S LETTER-BOX.

Correspondence on all subjects is invited, but we cannot in any way be responsible for the opinions expressed by our correspondents, who must give their name and address as a guarantee of good faith, but not necessarily for publication. Correspondents are reminded that brevity of style and conciseness of statement greatly facilitate early insertion.

### State Assistance to Voluntary Hospitals. Free Donations from Income- and Super-Taxes.

*To the Editor of* THE HOSPITAL.

SIR,—As Chairman of a Metropolitan hospital with 173 beds I am concerned at the ever-increasing cost of maintenance, and there is no doubt that all the other hospitals are afflicted in the same way.

There is a general feeling that assistance should come from the State, and that such assistance should not involve control of the management of the voluntary hospitals; but I have seen no definite proposal as to the form such assistance should take, and I therefore venture to make the suggestion that it should take the form of remission of income- and super-tax on all donations to recognised hospitals.

At the present time a gift to a hospital by an individual in the enjoyment of an income of from £7,000 to £9,000 a year has to bear taxation of 9s. 6d. in the £, or, in other words, if a man gives £200 to a hospital he must earn £380 to enable him to do so, owing to the amount of income- and super-tax he has to pay, and of course if his income is more than £9,000 a year, his earnings must be even greater in proportion as the rate of super-tax rises. I think this is a great deterrent, for if a man feels that the institution he wishes to help is practically getting little more than half of the result of his personal effort, it makes him less inclined to make the effort.

Now if an individual were permitted to treat donations or subscriptions to a recognised hospital as a deduction from his income, both for Schedule D and super-tax, I think the hospitals would benefit largely, and the State might quite likely be saved from having to contribute to their upkeep, with all the costly machinery that would probably be instituted in order to guard such contributions from abuse, and the loss of revenue by the suggested remission of the income- and super-tax would be far less than the cost of directly contributing to upkeep of the hospitals.

In the case of smaller incomes the loss to the State would be less, and in many cases doubtless there would be no loss of revenue at all, for many individuals would fail to reclaim the tax on their donations.

That hospitals should be free of income-tax is already recognised so far as their income is derived from invested funds, and it is not a great step to free from this impost the contributions of individuals to the support of the voluntary hospitals.

The need of some assistance is urgent, for though old subscribers loyally keep up their subscriptions and even increase them, there is an absence of new support, and it is evident that possessors of newly-acquired wealth have not yet realised the responsibilities of wealth and prefer to spend it on expensive luxuries rather than bear their fair share in supporting such national necessities as voluntary hospitals. Moreover, the borrowing powers of most hospitals are rapidly dwindling, not only on account of increased expenditure, but also on account of the capital depreciation of all so-called "gilt-edged" securities.

The anxiety of those responsible for the management of large hospitals is daily increasing, and if you will kindly publish this letter I venture to hope that it may lead to the expression of public opinion in favour of relief in the direction I suggest, without which I fear there is little hope of action on the part of the Treasury.—I am, yours truly, R. H. CAIRD,

Chairman of the Board of Management.

London Homœopathic Hospital, W.C. 1,

April 8, 1920.

